# Severe Acute Respiratory Syndrome Coronavirus 2 Seropositivity among Healthcare Personnel in Hospitals and Nursing Homes, Rhode Island, USA, July–August 2020

**DOI:** 10.3201/eid2703.204508

**Published:** 2021-03

**Authors:** Lara J. Akinbami, Philip A. Chan, Nga Vuong, Samira Sami, Dawn Lewis, Philip E. Sheridan, Susan L. Lukacs, Lisa Mackey, Lisa A. Grohskopf, Anita Patel, Lyle R. Petersen

**Affiliations:** Centers for Disease Control and Prevention, Hyattsville, Maryland, USA (L. Akinbami, S.L. Lukacs);; US Public Health Service, Rockville, Maryland, USA (L. Akinbami, S.L. Lukacs, L.A. Grohskopf);; Rhode Island Department of Health, Providence, Rhode Island, USA (P.A. Chan, D. Lewis, P.E. Sheridan);; Centers for Disease Control and Prevention, Fort Collins, Colorado, USA (N. Vuong, L. Mackey, L.R. Petersen);; Centers for Disease Control and Prevention, Atlanta, Georgia, USA (S. Sami, L.A. Grohskopf, A. Patel)

**Keywords:** SARS-CoV-2, seroprevalence, nursing home, long term care facility, hospital, healthcare personnel, Rhode Island, respiratory infections, severe acute respiratory syndrome coronavirus 2, SARS, coronavirus disease, zoonoses, viruses, coronavirus, United States

## Abstract

Healthcare personnel are recognized to be at higher risk for infection with severe acute respiratory syndrome coronavirus 2. We conducted a serologic survey in 15 hospitals and 56 nursing homes across Rhode Island, USA, during July 17–August 28, 2020. Overall seropositivity among 9,863 healthcare personnel was 4.6% (95% CI 4.2%–5.0%) but varied 4-fold between hospital personnel (3.1%, 95% CI 2.7%–3.5%) and nursing home personnel (13.1%, 95% CI 11.5%–14.9%). Within nursing homes, prevalence was highest among personnel working in coronavirus disease units (24.1%; 95% CI 20.6%–27.8%). Adjusted analysis showed that in hospitals, nurses and receptionists/medical assistants had a higher likelihood of seropositivity than physicians. In nursing homes, nursing assistants and social workers/case managers had higher likelihoods of seropositivity than occupational/physical/speech therapists. Nursing home personnel in all occupations had elevated seropositivity compared with hospital counterparts. Additional mitigation strategies are needed to protect nursing home personnel from infection, regardless of occupation.

Healthcare personnel face higher risk of infection during the coronavirus disease (COVID-19) pandemic because of their essential role in identifying and treating persons affected (*1*,*2*). Although essential workers in many occupations have higher risk of infection because of face-to-face interaction with the public, personnel in hospitals and nursing homes have more frequent and prolonged contact with persons known to be infected with severe acute respiratory syndrome coronavirus 2 (SARS-CoV-2).

Hospitals and nursing homes are potential hotspots of infection transmission. Hospital personnel conduct activities ranging from infection screening to administering advanced life support measures and may be exposed to patients with high viral loads (*3*). Infection risk can be exacerbated by shortages in personal protective equipment (PPE) and other resources, including staff (*4*,*5*). Nursing homes have been referred to as “ground zero” (*6*) of the pandemic because resident deaths have contributed disproportionately to overall COVID-19 mortality (*2*,*7*). Several factors may increase intrafacility transmission, including residents with risk factors for severe COVID-19 disease and prolonged viral shedding (e.g., advanced age, underlying conditions), a large proportion of asymptomatic infections, and new resource constraints alongside long-standing challenges (*8*–*11*). Assessing SARS-CoV-2 seropositivity among hospital and nursing home personnel may reveal risk factors that can be addressed through additional interventions. Community transmission has been identified as a primary determinant of transmission in both nursing homes and hospitals (*12*,*13*), but the relative impact in each of these settings has not been simultaneously compared.

The Rhode Island Department of Health (RIDOH) and the US Centers for Disease Control and Prevention (CDC) collaborated on a serologic survey of personnel in hospitals, nursing homes, and first responder agencies (e.g., fire, law enforcement) across Rhode Island. As of July 17, 2020, when the survey was initiated, there were >17,700 persons positive for COVID-19 in Rhode Island, of whom 2,675 were nursing home residents and 1,210 nursing home staff, and just more than 1,000 deaths, most among nursing home residents (*14*). Because of the disproportionate impact on nursing homes, we made an added effort to include as many nursing home facilities as possible in the survey. This analysis compares SARS-CoV-2 seroprevalence among nursing homes and hospital personnel and assesses characteristics and factors related to seropositivity.

## Methods

The serologic survey was conducted throughout Rhode Island during July 17–August 28, 2020. RIDOH performed outreach to all agencies to encourage participation. The protocol was reviewed by CDC human subjects research officials, who determined that the activity was public health surveillance as defined in 45 CFR 46 (*15*). Participation was voluntary, results were not shared with employers, and CDC did not have access to personally identifying information.

RIDOH provided participating agencies with study information and a link to the secure web-based survey to distribute to employees (Appendix Table 1). Upon completing the screening and questionnaire on a personal device, participants received information about blood collection events at their workplace or nearby facility. Each participant provided 10–15 mL of blood using standard venipuncture techniques. Centrifuged serum samples were transferred to a central laboratory for SARS-CoV-2 antibody testing using the ORTHO Clinical Diagnostics VITROS Immunodiagnostic Products Anti-SARS-CoV-2 IgG Test (https://www.orthoclinicaldiagnostics.com). The emergency use authorization data submitted to the US Food and Drug Administration indicated that this test measures IgG directed at the S1 domain of the spike protein with a sensitivity of 90% and a specificity of 100% (*16*). Results were reported to participants as negative (signal-to-cutoff ratio <1.0), positive (>1.0), or lack of valid result.

**Table 1:** SARS-CoV-2 seropositivity among hospital and nursing home personnel, by demographic characteristics, Rhode Island, USA, July–August 2020*

Characteristic	**Hospital**				**Nursing home**		
	No. (%)	Seropositive, no.	Seropositive, % (95% CI)		No. (%)	Seropositive, no.	Seropositive, % (95% CI)
Total	8,370 (100)	256	3.1 (2.7–3.5)		1,494 (100)	196	13.1 (11.5–15.0)
Age group, y							
18–24	275 (3.3)	21	7.6 (4.8–11.4)		68 (4.6)	7	10.3 (4.2–20.1)
25–34	1,987 (23.7)	71	3.6 (2.8–4.5)		254 (17.0)	37	14.6 (10.5–19.5)
35–44	1,874 (22.4)	56	3.0 (2.3–3.9)		328 (22.0)	45	13.7 (10.2–17.9)
45–59	2,890 (34.5)	81	2.8 (2.2–3.5)		569 (38.1)	78	13.7 (11.0–16.8)
60–64	896 (10.7)	22	2.5 (1.6–3.7)		170 (11.4)	20	11.8 (7.3–17.6)
>65	448 (5.4)	5	1.1 (0.4–2.6)		105 (7.0)	9	8.6 (4.0–15.7)
Sex							
M	1,582 (18.9)	44	2.8 (2.0–3.7)		227 (15.2)	39	17.2 (12.5–22.7)
F	6,788 (81.1)	212	3.1 (2.7–3.6)		1,267 (84.8)	157	12.4 (10.6–14.3)
Race/ethnicity							
Non-Hispanic White	6,829 (81.6)	182	2.7 (2.3–3.1)		1,165 (78.0)	119	10.2 (8.5–12.1)
Non-Hispanic Black	284 (3.4)	20	7.0 (4.4–10.7)		87 (5.8)	24	27.6 (18.5–38.2)
Non-Hispanic Asian	316 (3.8)	10	3.2 (1.5–5.7)		28 (1.9)	6	21.4 (8.3–41.0)
Hispanic	554 (6.6)	31	5.6 (3.8–7.9)		130 (8.7)	28	21.5 (14.8–29.6)
Other†	191 (2.3)	11	5.8 (2.9–10.1)		40 (2.7)	8	20.0 (9.1–36.7)
Decline	196 (2.3)	2	1.0 (0.1–3.6)		44 (2.9)	11	25.0 (13.2–40.3)
Housing							
Single family	6,924 (82.7)	204	3.0 (2.6–3.4)		1,136 (76.0)	131	11.5 (9.7–13.5)
Multiunit	1,446 (17.3)	52	3.6 (2.7–4.7)		358 (24.0)	65	18.2 (14.3–22.6)

*SARS-CoV-2, severe acute respiratory syndrome coronavirus 2. †Other race/ethnicity includes non-Hispanic Native Hawaiian and other Pacific Islander, non-Hispanic American Indian and Alaska Native, and participants who indicated other non-Hispanic race.

A total of 11,987 participants ≥18 years of age consented to phlebotomy and reported no new symptoms of cough, shortness of breath, fever, change in sense of taste/smell, or positive test for SARS-CoV-2 by reverse transcription PCR (RT-PCR) in the 2 weeks before survey participation. Seven were excluded for lack of valid serologic test result because of lipemia or insufficient sample volume and 1,860 did not work in either a hospital (inpatient units and/or ambulatory clinics) or nursing home. Of the remaining 10,120 participants, 9,863 had occupations in direct patient care and support (Appendix Table 2) and were included in this analysis.

**Table 2:** SARS-CoV-2 seropositivity among hospital and nursing home personnel, by occupation and work location, Rhode Island, USA, July–August 2020*

Category	**Hospital**				**Nursing home**		
	No.	Seropositive, no.	Seropositive, % (95% CI)		No.	Seropositive, no.	Seropositive, % (95% CI)
Occupation							
Administrative/office staff/clerk	903	19	2.1 (1.3–3.3)		200	11	5.5 (2.8–9.6)
Diagnostic Imaging	369	11	3.0 (1.5–5.3)		0	NA	NA
Dietician/dietary services	135	3	2.2 (0.5–6.4)		114	10	8.8 (4.3–1.6)
Engineer/maintenance	108	2	1.9 (0.2–6.5)		26	6	23.1 (9.0–43.7)
Environmental services/cleaning	114	3	2.6 (0.6–7.5)		69	9	13.0 (6.1–23.3)
Laboratory technologist/technician	281	4	1.4 (0.4–3.6)		0	NA	NA
Nurse	2,733	114	4.2 (3.5–5.0)		413	63	15.3 (11.9–19.1)
Nurse assistant	392	23	5.9 (3.8–8.7)		296	59	19.9 (15.5–24.9)
Occupational/physical/speech therapist	283	8	2.8 (1.2–5.5)		163	16	9.8 (5.7–15.5)
Other healthcare	573	12	2.1 (1.1–3.6)		65	4	6.2 (1.7–15.0)
Pharmacist/pharmacist assistant	256	7	2.7 (1.1–5.6)		5	2	40.0 (5.3– 85.3)
Physician	1,001	22	2.2 (1.4–3.3)		10	0	0.0
Physician assistant	100	1	1.0 (0.0–5.5)		0	NA	NA
Receptionist/medical assistant	296	12	4.1 (2.1–7.0)		15	1	6.7 (0.2–32.0)
Social worker/case manager/counselor	432	7	1.6 (0.1–3.3)		46	10	21.7 (11.0–36.4)
Supervisor/manager	393	8	2.0 (0.9–4.0)		72	5	6.9 (2.3–15.5)
Workplace†							
Administrative office	1,132	21	1.9 (1.2–2.8)		218	12	5.5 (2.9–9.4)
Ambulatory healthcare/dental office	2,122	48	2.3 (1.7–3.0)		NA	NA	NA
Hospital COVID-19 unit	1,435	72	5.0 (4.0–6.3)		NA	NA	NA
Hospital general inpatient unit	3,752	138	3.7 (3.1–4.3)		NA	NA	NA
Hospital intensive care unit	1,250	37	3.0 (2.1–4.1)		NA	NA	NA
Hospital surgical unit	1,234	31	2.5 (1.7–3.6)		NA	NA	NA
Hospital emergency department	288	7	2.4 (1.0–4.9)		NA	NA	NA
Other hospital location	963	20	2.1 (1.3– 3.2)		NA	NA	NA
Nursing home COVID-19 unit	NA	NA	NA		565	136	24.1 (20.6–27.8)
Nursing home non–COVID-19 unit	NA	NA	NA		1,088	111	10.2 (8.5–12.2)

*Gray shading indicates nursing home occupation categories that had a sample size <30 and were combined into an other nursing home category, with a combined n = 56, percent seropositive 16.1% (7.6%–28.3%). COVID-19, coronavirus disease; NA, not applicable; SARS-CoV-2, severe acute respiratory syndrome coronavirus 2. †Work location categories are not mutually exclusive: 27.3% of participants reported working in >1 workplace. Hospital and nursing home participants also reported working in other workplaces not shown in the table: corrections facilities (n = 16), Rhode Island Department of Health (n = 4), emergency medical services (n = 15), fire department (n = 6), law enforcement (n = 1), Rhode Island emergency management (n = 8), Rhode Island alternative hospital setup site (n = 14), Rhode Island remote COVID-19 testing site (n = 21), Rhode Island state warehouse (n = 1), or Rhode Island traffic and perimeter control (n = 1). Some worked in facilities in the other agency category; that is, 84 hospital personnel also worked in nursing home COVID-19 and non–COVID-19 units, and 34 nursing home personnel also worked in hospital COVID-19 units and general inpatient units.

We calculated seropositivity (percent positive for SARS-CoV-2 antibodies) overall and for subgroups. We estimated exact Clopper-Pearson 95% CIs and assessed significant statistical differences by evaluating nonoverlapping 95% CI or χ^2^ tests for categorical variables and Cochran-Armitage trend tests for ordinal variables (2-sided with α = 0.05).

We classified participants who reported race/ethnicity as non-Hispanic Native Hawaiian or other Pacific Islander, non-Hispanic American Indian or Alaska Native, or other race as other race (n = 231, 2.3%) and those who declined to specify race/ethnicity as declined (n = 240, 2.4%). We stratified analyses by primary agency selected by participants: hospital or nursing home. Participants could then choose one or more specific workplaces from a precategorized list or free-text workplaces not listed. Hospital emergency department was inadvertently omitted from the response categories for specific workplace but was included in the analysis based on free-text responses. Some hospital and nursing home participants reported working in additional settings that were not the focus of the analysis (e.g., emergency medical services) or in the other agency type (e.g., 1% of hospital and 2% of nursing home personnel worked in both hospital and nursing home settings). These participants were retained in the analysis, but these other workplaces were reported infrequently and are not shown separately. A precategorized list and free-text option were also provided for occupation. Prespecified categories with low frequencies were combined (Appendix Table 2). Among nursing home occupations, 4 with low sample size were combined (other nursing home: engineer/maintenance staff, pharmacist, receptionist/medical assistant, and physician, n = 56). Analyzing workplace and occupation simultaneously resulted in small sample sizes. Only occupation/workplace groups with sample size >20 or with absolute 95% CI width >30% were shown to ensure estimate reliability (*17*). Each workplace was represented as a separate dichotomous variable to allow modeling of non–mutually exclusive categories.

Participants reported the frequency at which they performed aerosol-generating procedures; if they needed complete PPE, as defined by CDC recommendations by occupation and patient contact; if, since March 1, they ever used PPE shortage protocols (extended use, reuse, or both); if they lacked specific PPE components when in contact with a person with suspected/confirmed COVID-19 in the workplace; and if they received training in the previous year on PPE donning/doffing techniques. Participants also reported whether their work involved in-person interaction with the community, patients, or both and if they were exposed (spent >10 minutes within 6 feet) to any COVID-19 positive co-workers, household members, patients, or other persons.

We used generalized estimating equations to model likelihood of seropositivity, accounting for clustering by facility (15 hospitals and 56 nursing homes, using an independence correlation structure). PPE variables had a common category (never use PPE) and were thus collinear. Therefore, only PPE shortage protocol use was included in the model, given evidence that shortages may contribute to transmission (*12*). Similarly, questions assessing use of individual PPE components had a common category, not applicable. Of these, only use of an N95/powered air-purifying respirator (PAPR) was included in the model, because it had an unadjusted association with seroprevalence. For hospital occupations, physicians were the reference group for comparability to a previous study (*18*). There were not enough physicians in nursing homes to categorize separately, so occupational/physical/speech therapists were the reference group for nursing homes. No interaction terms were explored. We used SAS 9.4 software (SAS Institute, https://www.sas.com) for all analyses.

## Results

**Figure 1:**
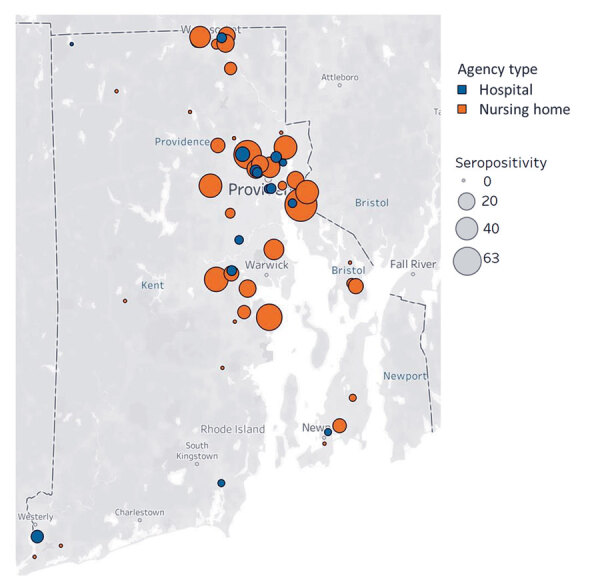
Seropositivity for severe acute respiratory syndrome coronavirus 2 among hospital and nursing home personnel, by facility, Rhode Island, USA, July–August 2020. Map based on average of longitude and average of latitude. Marker size is proportional to facility-level seroprevalence. Facilities with participant sample size <10 are not shown.

Overall seropositivity for 9,863 participants was 4.6% (95% CI 4.2%–5.0%) but differed between hospital personnel (3.1%; 95% CI 2.7%–3.5%) and nursing home personnel (13.1%; 95% CI 11.5%–15.0%) (Table 1). Generally, we found higher facility-level seropositivity in nursing homes than in hospitals, as well as lower or 0% seropositivity in facilities in rural western Rhode Island (Figure 1). Demographic characteristics were similar between hospital and nursing home personnel, but some seropositivity patterns differed. Seropositivity was highest among hospital personnel 18–24 years of age, but there were no age differences among nursing home personnel (p = 0.64 by χ^2^ test). For both groups, there were no differences by sex (p>0.05), and Hispanic and non-Hispanic Black personnel had higher seropositivity compared with non-Hispanic White personnel (pairwise p<0.001 for both groups). Among nursing home personnel, those who lived in multiunit housing had higher seroprevalence than those in single-family housing (p = 0.001).

**Figure 2:**
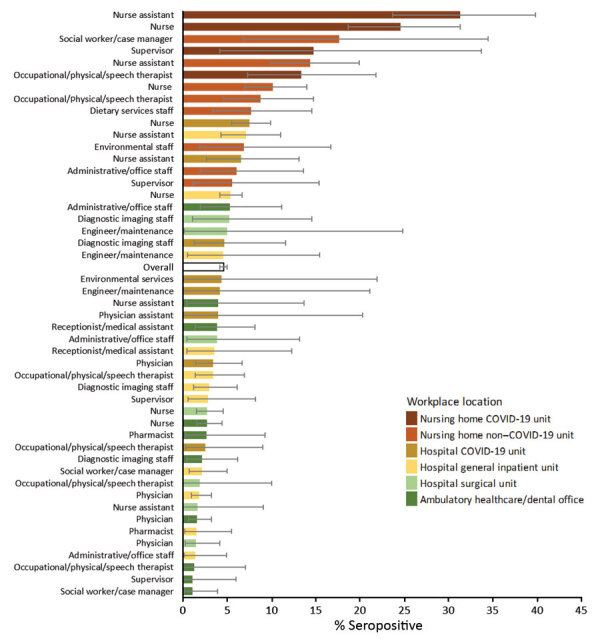
Seropositivity for severe acute respiratory syndrome coronavirus 2 among hospital and nursing home personnel, by selected workplace and occupation, Rhode Island, USA, July–August 2020. Error bars indicate 95% CIs. Workplace/occupation categories are not mutually exclusive: 27.3% of participants indicated >1 workplace. Occupations not included in the figure had 0% seroprevalence, sample size below n = 20, or absolute CI width >0.30 (unreliable estimate). Other healthcare category also not included. COVID-19, coronavirus disease.

Among hospital personnel, nurse assistants had higher seropositivity (5.9%, 95% CI 3.8%–8.7%) than the overall hospital level of 3.1% (Table 2). Among nursing home personnel, nurse assistants had higher seropositivity (19.9%, 95% CI 15.5%–24.9%) than the overall nursing home level of 13.1%. Overall, 27.3% of participants reported working at >1 workplace. Among hospital personnel, seropositivity was higher among those working in hospital COVID-19 units (5.0%, 95% CI 4.0%–6.3%) than the overall hospital level. Among nursing home personnel, those working in nursing home COVID-19 units had higher seropositivity (24.1%, 95% CI 20.6%–27.8%) than the overall nursing home level. Figure 2 shows workplace and occupation together in non–mutually exclusive categories. Occupation/workplace groups with seroprevalence significantly elevated above the overall level of 4.6% included nurse assistants (31.4%, 95% CI 23.7%–39.9%), nurses (24.6%, 95% CI 18.7%–31.4%), and occupational therapists (13.4%, 95% CI 7.3%–21.8%) who worked in nursing home COVID-19 units; social workers/case managers (17.7%, 95% CI 6.8%–34.5%), nurse assistants (14.4%, 95% CI 10.0%–20.0%), and nurses (10.2%, 95% CI 7.1%–14.0%) who worked in nursing home non–COVID-19 units; and nurses (7.5%, 95% CI 5.5%–9.9%) who worked in hospital COVID-19 units. Across all occupational groups, seropositivity was higher for those who worked in nursing homes compared with those with the same occupation in hospitals.

Among hospital personnel, 27.2% of those exposed to a household member who tested positive for COVID-19 were seropositive versus 2.4% of those unexposed (Table 3). For nursing home personnel, 54.0% of those exposed to a household member with COVID-19 were seropositive versus 10.9% of those unexposed. For both hospital and nursing home personnel, exposure versus no exposure to a co-worker was associated with higher seropositivity, as was exposure to a patient (with or without PPE use) and exposure to some other person. Seropositivity was higher among personnel with community or patient interaction as part of work responsibilities compared with those without for both hospital (3.2% vs. 0.9%) and nursing home personnel (13.7% vs. 7.3%).

**Table 3:** SARS-CoV-2 seropositivity among hospital and nursing home personnel, by exposure to persons testing positive for COVID-19 and in-person interaction in the workplace, Rhode Island, USA, July–August 2020*

Question	Hospital				Nursing home		
	No.	Seropositive, no.	Seropositive, % (95% CI)		No.	Seropositive, no.	Seropositive, % (95% CI)
Exposed to COVID-19–positive co-worker?							
Exposed	2,070	122	5.9 (4.9–7.0)		550	113	20.6 (17.2–24.2)
Not exposed/don't know	6,299	134	2.1 (1.8–2.5)		944	83	8.8 (7.1–10.8)
Exposed to COVID-19–positive household member?							
Exposed	213	58	27.2 (21.8–33.7)		76	41	54.0 (42.1–65.5)
Not exposed/don't know	8,156	198	2.4 (2.1–2.8)		1,418	155	10.9 (9.4–12.7)
Exposed to COVID-19**–**positive patient?							
Exposed while not wearing PPE	1,317	60	4.6 (3.5–5.8)		173	28	16.2 (11.0–22.5)
Exposed while wearing PPE	2,630	108	4.1 (3.4–4.9)		498	119	23.9 (20.2–27.9)
Not exposed/don't know	4,422	88	2.0 (1.6–2.5)		823	49	6.0 (4.4–7.8)
Exposed to other COVID-19**–**positive person?							
Exposed	827	67	8.1 (6.3–10.2)		163	54	33.1 (26.0–40.9)
Not exposed/don't know	7,542	189	2.5 (2.2–2.9)		1,331	142	10.7 (9.1–12.5)
In-person interaction with public/patients in the workplace?							
Work involves in-person interaction	7,795	251	3.2 (2.8–3.6)		1,370	187	13.7 (11.9–15.6)
No in-person interaction	574	5	0.9 (0.3–2.0)		124	9	7.3 (3.4–13.3)

*Exposure defined as being within 6 feet for at least 10 min. COVID-19, coronavirus disease; PPE, personal protective equipment; SARS-CoV-2, severe acute respiratory syndrome coronavirus 2.

**Figure 3:**
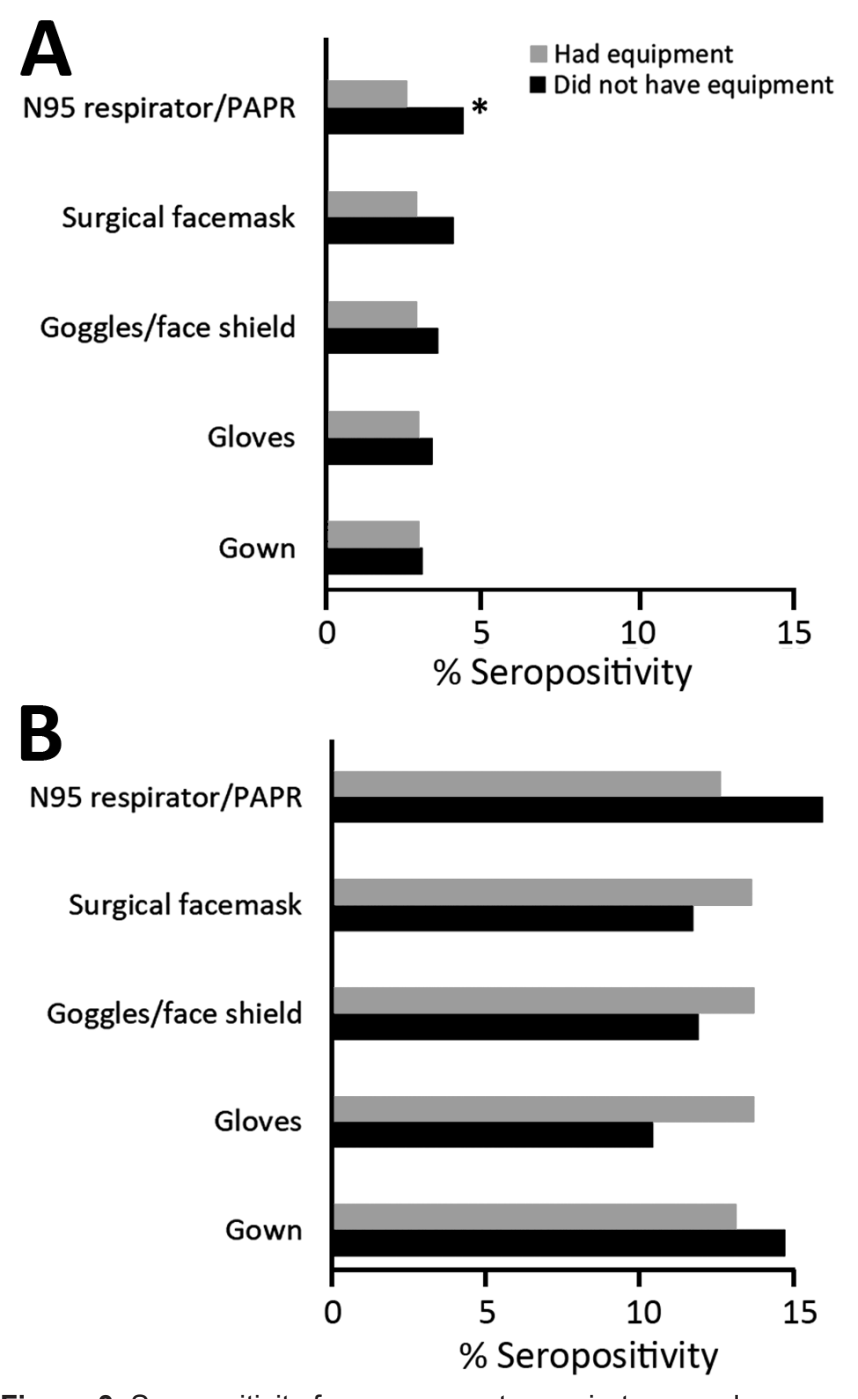
Seropositivity for severe acute respiratory syndrome coronavirus 2 among hospital and nursing home personnel, by having/not having specific PPE, Rhode Island, USA, July–August 2020. Excludes participants who reported no PPE use (19.6% of those in hospital settings, seropositivity 3.4%; 12.4% of those in nursing home settings, seropositivity 12.4%). Asterisk (*) indicates statistically significant difference (p<0.05 by χ^2^ test). PPE, personal protective equipment.

For both hospital and nursing home personnel, we found a significant linear trend of increasing seropositivity with greater procedure frequency of performing aerosol-generating procedures (Table 4). For both groups, seropositivity decreased with decreasing frequency of needing complete PPE. Among hospital personnel, those who reported no shortage of PPE had higher seropositivity than those who reused PPE (p = 0.006). Among nursing home personnel, there were no significant differences in seropositivity between those who reported no PPE shortages and those who reported extended use, reuse, or both. Among all personnel, there were no differences in seroprevalence between those who received PPE donning/doffing training versus those with no training (p>0.05 by χ^2^ test). For each equipment type, there were no differences in seropositivity between those who reported having versus not having a specific PPE component, with one exception: hospital personnel who did not have an N95 respirator/PAPR were more likely to be seropositive than those who had this equipment (4.4% vs. 2.6%) (Figure 3).

**Table 4:** SARS-CoV-2 seropositivity among hospital and nursing home personnel, by frequency of conducting aerosol-generating procedures frequency and use of PPE, Rhode Island, USA, July–August 2020*

Characteristic	Hospital				Nursing home		
	No.	Seropositive, no.	Seropositive, % (95% CI)		No.	Seropositive, no.	Seropositive, % (95% CI)
Aerosol-generating procedure frequency							
0 times per shift per week	4,121	108	2.6 (2.2–3.2)		858	93	10.8 (8.8–13.1)
1–5 times	1,679	62	3.7 (2.8–4.7)		114	25	21.9 (14.7–30.7)
6–10 times	380	22	5.8 (3.7–8.6)		36	7	19.4 (8.2–36.0)
11–25 times	277	11	4.0 (2.0–7.0)		23	4	17.4 (5.0–38.8)
>25 times	366	19	5.2 (3.2–8.0)		41	12	29.3 (16.1–45.5)
NA	1,546	34	2.2 (1.5–3.1)		422	55	13.0 (10.0–16.6)
PPE use							
Never use PPE	2,939	64	2.2 (1.7–2.8)		322	19	5.9 (3.6–9.1)
Used PPE and reported frequency of needing complete PPE							
Daily	1,809	66	3.7 (2.8–4.6)		632	125	19.8 (16.7–23.1)
Few times a week	1,860	75	4.0 (3.2–5.0)		332	42	12.7 (9.3–16.7)
Less than once a week	1,761	51	2.9 (2.2–3.8)		208	10	4.8 (2.3–8.7)
Use of PPE shortage protocol							
No shortage	511	25	4.9 (3.2–7.1)		238	28	11.8 (8.0–16.6)
Reuse	934	21	2.3 (1.4–3.4)		186	21	11.3 (7.1–16.7)
Extended use	1,341	42	3.1 (2.3–4.2)		253	45	17.8 (13.3–23.1)
Extended and reuse	2,644	104	3.9 (3.2–4.8)		495	83	16.8 (13.6–20.4)
Donning/doffing training in past year							
Yes	5,140	184	3.6 (3.1–4.1)		1,135	170	15.0 (13.0–17.2)
No	199	5	2.5 (0.8–5.8)		15	3	20.0 (4.3–48.1)
Don't know	91	3	3.3 (0.7–9.3)		22	4	18.2 (5.2–40.3)

*Significant linear trend of seropositivity with rising frequency of aerosol-generating procedures and decreasing frequency of needing complete PPE for hospital and nursing home settings (p<0.001 for all). NA, not applicable; PPE, personal protective equipment; SARS-CoV-2, severe acute respiratory syndrome coronavirus 2.

**Figure 4:**
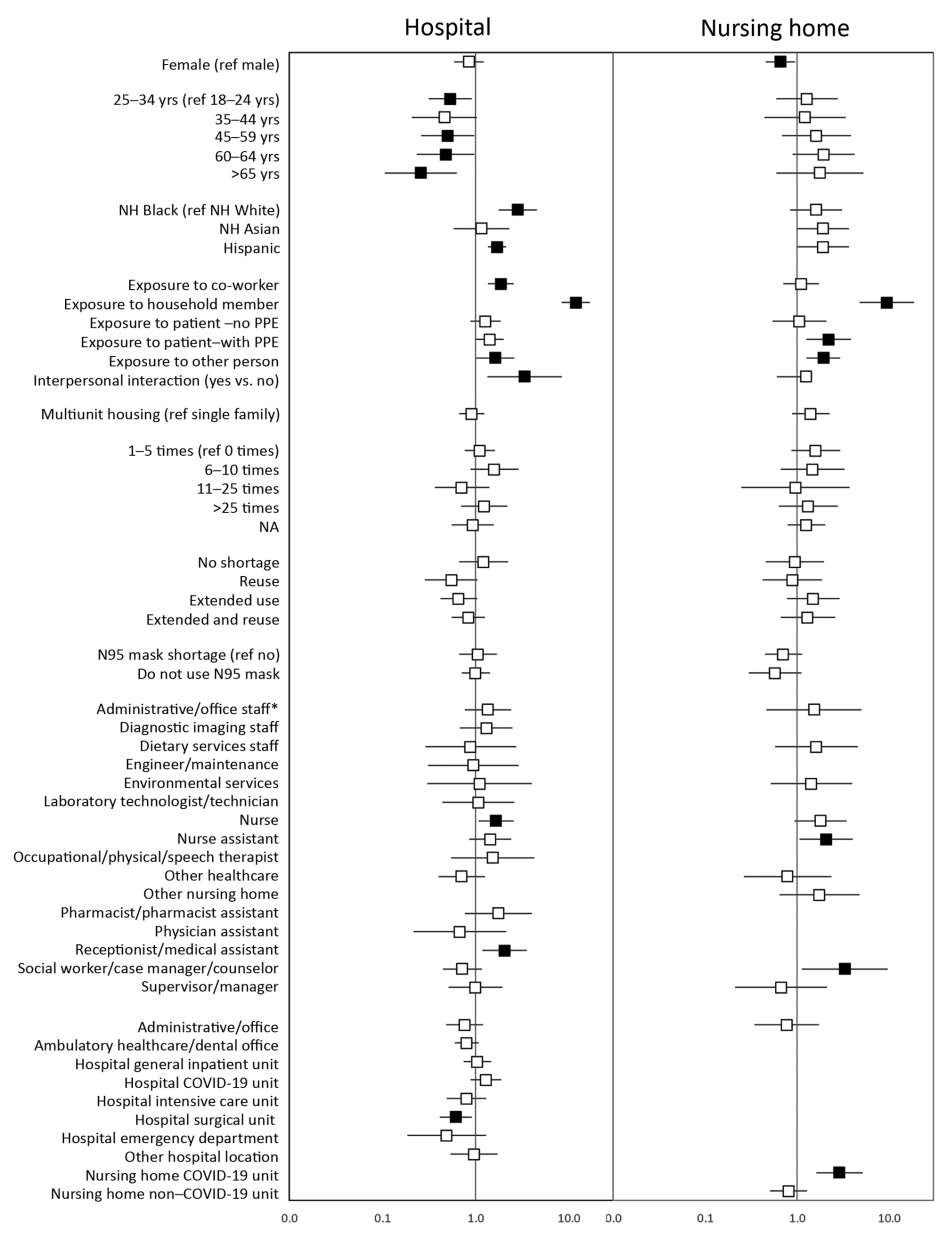
Adjusted odds ratios and 95% CIs for seropositivity, Rhode Island, USA, July–August 2020. The adjusted models were estimated using generalized estimating equations including all variables shown. Error bars indicate 95% CIs; black boxes denote adjusted odds ratios for which the 95% CI excludes 1.0. Workplace was represented by non–mutually exclusive dummy variables entered simultaneously into the model; the referent group for each workplace is not working in that specific workplace. Participants in workplaces with sample size <30 or with 0% seropositivity were included in the model but the workplace was not entered into the model. *For the hospital model, physicians were the referent occupation group. For the nursing home model, occupational/physical/speech therapists were the referent occupation group. Ref, referent; NH, non-Hispanic; PPE, personal protective equipment.

In adjusted models (Figure 4; Appendix Table 3), both hospital personnel (Figure 4, panel A) and nursing home personnel (Figure 4, panel B) with exposure to a household member with COVID-19 had the highest odds of being seropositive. Otherwise, seropositivity patterns diverged by facility type. For hospital personnel, older age compared with 18–24 years of age was associated with lower seropositivity and non-Hispanic Black and Hispanic race/ethnicity were associated with higher seropositivity. Among nursing home personnel, there was no significant pattern of seropositivity by age or race/ethnicity. Personnel with work responsibilities including face-to-face interaction with members of the community or patients had a higher likelihood of seropositivity among hospital but not nursing home personnel. Among hospital personnel, nurses and receptionists or medical assistants had a higher likelihood of being seropositive compared with physicians. Among nursing home personnel, nurse assistants and social workers or case managers had higher likelihood compared with occupational, physical, and speech therapists. Finally, hospital personnel working in surgical units had lower likelihood of being seropositive. There were no associations by frequency of aerosol-generating procedures, use of PPE shortage protocols, or not having or using an N95 respirator/PAPR among either hospital or nursing home personnel.

## Discussion

In this study, we compared SARS-CoV-2 seroprevalence among nursing home personnel to hospital personnel within 1 state. Nursing home personnel had a significantly higher seroprevalence (13.1%) than hospital personnel (3.1%), who had levels comparable to statewide seroprevalence of 2.8% based on commercial laboratory data as of August 2020 (*19*). High prevalence among nursing home personnel was observed across all occupations studied. A study analyzing Centers for Medicare and Medicaid Services facility-level data found that community COVID-19 prevalence was the strongest predictor of COVID-19 cases and deaths in nursing homes (*12*). In this study, the association between facility and community seroprevalence may hold, but with exaggerated SARS-CoV-2 transmission in nursing homes versus hospitals. SARS-CoV-2 seropositivity among nursing home COVID-19 unit personnel was nearly 5 times higher than among hospital-based COVID-19 unit personnel. Nursing home non–COVID-19 unit personnel had seropositivity nearly 3 times higher than hospital general inpatient unit personnel. As of November 17, 2020, all 85 Rhode Island nursing homes had reported >1 COVID-19 cases; weekly counts of new cases were approximately equal for nursing home residents and staff, at ≈185 each as of November 25, 2020, according to RIDOH SARS-CoV-2 surveillance. Nursing homes have been deemed tinderboxes because of a constellation of factors that may perpetuate transmission, including resident populations with risk factors for severe COVID-19 and prolonged viral shedding, residents who may be asymptomatic or have nonspecific symptoms of infection (e.g., increased confusion), shared caretakers between patients, chronic staffing shortages that may be exacerbated by worker illness, and lack of testing and PPE (*10*,*12*,*20*–*22*). In addition, suboptimal infection control practices have been noted in direct observation studies of nursing home personnel (*23*).

We found patterns among hospital and nursing home personnel that suggest both community- and workplace-acquired infection. In both settings, contact with a COVID-19–positive household member was the strongest risk factor for seropositivity. Adjusted odds ratios for seropositivity by age group and race/ethnicity reflected community patterns (*24*–*26*) among hospital personnel but not among nursing home personnel. Other studies have found that seroprevalence was correlated with local cumulative COVID-19 incidence in general (*12*,*13*,*18*). Workplace transmission is suggested by higher likelihood of seropositivity among occupations with frequent and prolonged patient contact or working in common areas: nurses and receptionists/medical assistants in hospital settings and nurse assistants and social workers/case managers in nursing homes. Similar findings were noted in other studies (*2*,*18*,*27*). In hospitals, interaction with patients and community members was associated with higher seropositivity than was having no interaction as part of work responsibilities. Finally, in agreement with results from other hospital studies, our study found lower seropositivity among personnel in a controlled environment: hospital surgical units (*5*,*18*). However, in nursing homes, workplace factors appeared to dominate community factors given the elevated risk across occupation and seroprevalence >4 times greater than community levels (2.8%). Intrafacility transmission was found in a study of 2 skilled nursing facilities in which viral strains within each facility were genetically more similar than between the 2 facilities or the community; within 1 facility, there were 2 genetically distinct strains, which suggested community introduction into the facility followed by intrafacility transmission (*27*). That is, this group of studies suggest that community introduction into nursing homes may result in higher level of intrafacility transmission compared with hospital settings.

In at least 2 ways, the higher seroprevalence among nursing home COVID-19 unit personnel could have been partially driven by cohorting residents. First, even if the probability of transmission in facilities were equal, a higher percentage of infectious patients and residents in COVID-19 units would result in a greater number of transmitted infections. Second, if previously infected staff were assigned to COVID-19 units, seroprevalence among facility staff would be increased through staffing decisions rather than transmission. Without longitudinal or genotyping data, it is not possible to disentangle intrafacility transmission. Staff in Rhode Island were rarely transferred between facilities according to past infection status. Two facilities designated as COVID-19 facilities accepted infected residents, and the other 54 facilities cohorted patients within the facility or transferred residents to other facilities with COVID-19 units. No data were gathered on staff transfers within facilities between COVID-19 and non–COVID-19 units. Despite these gaps in fully understanding transmission, seroprevalence was still greatly elevated in nursing homes compared with hospitals among both COVID-19 and non–COVID-19 unit personnel.

Unadjusted analyses showed that those with daily requirements for complete PPE were more likely to be seropositive for both groups. However, there were no significant adjusted associations between seropositivity and frequency of requirement for complete PPE or PPE shortage protocol use. These findings suggest that PPE use was likely a marker for increased occupational risk (i.e., frequent close contact with infected patients or residents) and that personnel with the most frequent or intense patient contact may have received priority for PPE supplies or that PPE shortages did not have a major role in transmission in this study. More detailed studies are necessary to disentangle the complex factors surrounding PPE use.

Limitations include the cross-sectional study design. Patient or resident infection status was not ascertained. Infection timing relative to different exposures is unknown. For example, it is unknown whether participants who reported exposure to a COVID-19 positive household member were infected by that contact or introduced the infection into the household. Similarly, among seropositive participants who reported working in >1 workplace, it is not possible to ascertain their contribution, if any, to transmission between facilities. Furthermore, seroprevalence is a cumulative measure; antibody responses are reported to persist for >4 months (*28*). The extent to which seroprevalence was related to exposures early in the pandemic, when PPE shortages were more acute and infection control measures were still being developed, is unknown. Participation was voluntary among a convenience sample, so representativeness of the population is unknown. However, 56 of 85 nursing homes in Rhode Island were included and seropositivity among nursing home participants was related to resident and staff case counts in facilities, with higher seropositivity with rising quartile of case counts (Appendix Table 4). No information was collected about other possible exposures, such as travel and commuting (e.g., use of public transportation). In addition, there could be uncontrolled confounding, including factors related to other socioeconomic factors, such as less flexibility for household members to telework or otherwise reduce occupational exposures. Strengths included a large sample size that allowed stable estimates among subgroups.

This study highlights the increased risk among nursing home personnel for SARS-CoV-2 infection compared with hospital personnel. Although this study was not designed to pinpoint mechanisms underlying the higher seroprevalence among nursing home personnel, 2 patterns strongly suggest that additional workplace protections may mitigate risk in this setting: the elevated risk among all nursing home occupations compared with hospital counterparts and the weaker signals of community transmission among nursing home settings (i.e., no association between age group and race/ethnicity with seropositivity). Continued attention to adherence with current infection control recommendations (e.g., PPE use, handwashing) and ensuring adequate testing, equipment, training, and staffing are the foundations for bolstering the safety of nursing home personnel (*22*,*23*,*29*).

Appendix Additional information about the study of SARS-CoV-2 antibody seroprevalence among healthcare personnel in hospitals and nursing homes, Rhode Island, USA, July–August 2020.
