# Supplementary material for: Severe Acute Respiratory Syndrome Coronavirus 2 Seropositivity among Healthcare Personnel in Hospitals and Nursing Homes, Rhode Island, USA, July–August 2020
Source: Emerg Infect Dis. 2021 Mar;27(3):823–34. doi: 10.3201/eid2703.204508 (PMC7920685; doi:10.3201/eid2703.204508)
Supplement: Appendix — Additional information about the study of SARS-CoV-2 antibody seroprevalence among healthcare personnel in hospitals and nursing homes, Rhode Island, USA, July–August 2020. [file 20-4508-Techapp-s1.pdf]

Article DOI: https://doi.org/10.3201/eid2703.204508

# Severe Acute Respiratory Syndrome Coronavirus 2 Seropositivity among Healthcare Personnel in Hospitals and Nursing Homes, Rhode Island, USA, July–August 2020

## Appendix

**Appendix Table 1.** Questionnaire administered to respondent healthcare personnel, Rhode Island, USA, July–August 2020

| Section | Question/item | Response categories |
|---|---|---|
| 1. | Name | First<br>Last |
| 2. | Home address | Street<br>City<br>State<br>Zip |
| 3.1 | Phone number (mobile) | Area code, phone number |
| 3.2 | Verify phone number (mobile) | Area code, phone number |
| 4.1 | Email address | (fill) |
| 4.2 | Verify email address | (fill) |
| 5. | Date of birth | MM/DD/YYYY |
| 6. | Sex at birth | Male<br>Female |
| 7. | Current gender | Man<br>Woman<br>Transgender man/trans man<br>Transgender woman/trans woman<br>Genderqueer/gender nonconforming neither exclusively male nor female<br>Other (fill)<br>Decline to answer |
| 8. | Sexual orientation | Gay<br>Straight<br>Bisexual<br>Something else/not sure<br>Decline to answer |
| 9. | Are you Hispanic or Latino/Latina? | Yes<br>No<br>Don't know<br>Decline to answer |
| 10. | What is your race?<br>(select all that apply) | White<br>Black/African American<br>Asian<br>American Indian or Alaska Native<br>Native Hawaiian or other Pacific Islander<br>Other<br>Decline to answer |
| Added | Please select your age group | 18–29 y<br>30–39 y<br>40–49 y<br>50–59 y<br>60–64 y<br>65–69 y<br>70–79 y |

| Section | Question/item | Response categories |
| --- | --- | --- |
| 11.1 | Select your primary agency or work category: | Hospital/healthcare agency<br>Fire agency<br>Law enforcement/police agency<br>EMS agency<br>Nursing home<br>Corrections (Go to 11.3)<br>RI National Guard (Go to 11.3)<br>RI Department of Health (Go to 11.3) |
| 11.2 | What is your primary work facility? | Hospitals (FULL LIST – 15 locations)<br>Fire (FULL LIST – Will split in half if needed – 47 locations)<br>Law Enforcement (FULL LIST – Will split in half if needed – 43 locations)<br>EMS (Split list in half – 79 locations)<br>Nursing homes (Split list in half – 85 locations) |
| 11.3 | Since March 1, did your work involve in-person interaction with members of the general community and/or patients? | Yes<br>No |
| 12.1 | What were your main workplaces since March 1st? (Select all that apply) | Administrative office (any facility)<br>Corrections—Intake<br>Corrections—Minimum Security<br>Corrections—Medium Security<br>Corrections—Maximum Security<br>Corrections—High Security<br>Corrections—Probation and Parole<br>Corrections—Women's Facility<br>Department of Health—Cannon Building<br>Department of Health—State Health Laboratory<br>Department of Health—Medical Examiner's Office<br>EMS- EMT/Paramedic<br>Fire Department<br>Law enforcement/Police<br>Healthcare—Ambulatory/Dental/Outpatient Clinic<br>Healthcare—Hospital general inpatient unit<br>Healthcare—Hospital COVID-19 unit<br>Healthcare—Hospital intensive care unit<br>Healthcare—Hospital surgical unit<br>Healthcare—Laboratory<br>Nursing home—COVID-19 unit<br>Nursing home—non–COVID-19 unit<br>RI Emergency Management Agency<br>RI alternate hospital setup site<br>RI remote COVID-19 testing site<br>RI state warehouses<br>Traffic and perimeter control<br>Other (any facility) |
| 12.2 | Which of the following best describes your occupation? | Administrative/business office staff/clerk<br>Clinical technician (e.g., cardiac, renal, surgical)<br>Clinical researcher/scientist<br>Corrections officer/staff<br>COVID-19 testing site staff<br>Dentist/dental hygienist/dental assistant<br>Diagnostic imagining staff<br>Dietician/dietary services staff<br>Dispatcher<br>Emergency medical technician (EMT)/paramedic<br>Engineer/maintenance/mechanic/repair staff<br>Environmental services/cleaning staff<br>Epidemiologist/infection control<br>Firefighter/fire inspector/fire marshal<br>Information/computer technologist<br>Laboratory technologist/technician<br>Law enforcement/police officer<br>Medical examiner/mortuary technician/forensic staff<br>Medical records/health information technician<br>Military servicemember<br>Nurse (e.g., CRNA, RN, LPN)<br>Nurse assistant (e.g., CNA, CSA, aide, medication technician)<br>Nurse midwife<br>Nurse practitioner<br>Occupational/physical/speech therapist |

| Section | Question/item | Response categories |
|---|---|---|
| | | Patient aide/technician<br>Pharmacist/pharmacist assistant<br>Phlebotomist/medical technician<br>Physician (e.g., MD, DO)<br>Physician assistant<br>Receptionist/medical assistant/patient registration<br>Recreation specialist/therapist<br>Respiratory therapist<br>Security guard<br>Social worker/case manager/counselor<br>Surgical technician/technologist<br>Supply chain/materials management (e.g., PPE)<br>Supervisor/manager<br>Other (fill) |
| 14. | Since March 1, on average, how many times per shift did you participate in any aerosol-generating procedures for suspected or confirmed COVID-19 patient(s)?<br>Examples:<br>Open suctioning of airways<br>Sputum induction<br>Cardiopulmonary resuscitation<br>Endotracheal intubation and extubation<br>Noninvasive ventilation (e.g., BiPAP, CPAP)<br>Bronchoscopy<br>Manual ventilation | More than 25 times<br>11- 25 times<br>6–10 times<br>1–5 times<br>0<br>Not applicable |
| 13.1 | CDC recommends "complete" PPE when in contact with suspected or confirmed COVID-19:<br>• Surgical face mask or respirator<br>• Goggles or face shield<br>• Gown<br>• Gloves<br>When aerosol generating or surgical procedures are performed, an N95 or higher-level respirator should be used instead of a surgical facemask.<br>On average since March 1, in your workplace, how often were you in a situation where you needed complete PPE? | Never (Go to 15.1)<br>Less than once a week<br>A few times a week<br>Every day |
| 13.2 | Since March 1, because of a PPE shortage, have you extended use of PPE (wearing the same PPE throughout an entire workday) or reused PPE at your workplace? | Yes, extended use of PPE<br>Yes, reused PPE<br>Yes, both extended use and reused<br>No shortage |
| 13.3 | At any time since March 1, which PPE did you NOT have/use when you were in contact with a person with suspected or confirmed COVID-19 at your workplace? (Select all that apply) | Gown<br>Gloves<br>Goggles/face shield<br>Surgical facemask<br>N95 respirator/PAPR/other respirator<br>NA |
| 13.4 | Have you been trained on how to properly put on and take off (don and doff) PPE in the past year? | Yes<br>No<br>Don't know |
| 15.1 | Did you spend more than 10 min within 6 feet of a co-worker who tested positive for COVID-19? | Yes<br>No<br>Don't know |
| 15.2 | Did you spend more than 10 min within 6 feet of a household member who tested positive for COVID-19? | Yes<br>No<br>Don't know |
| 15.3 | Did you spend more than 10 min within 6 feet of a patient who tested positive for COVID-19? | Yes, with appropriate PPE at all times<br>Yes, without appropriate PPE at least once<br>No<br>Don't know |

| Section | Question/item | Response categories |
|---|---|---|
| 15.4 | Did you spend more than 10 min within 6 feet of any other person who tested positive for COVID-19? | Yes<br>No<br>Don’t know |
| PREAMBLE:<br>Next, we will be asking you about two different types of testing—testing for current COVID-19 infection or testing for past infection (antibody). | | |
| 16.1 | Thinking about COVID-19, how many times were you tested for infection using a nasal, throat, or saliva sample? | [indicate 0 if none] (if 0 go to 17.1) |
| 16.2 | Have you ever had a positive nasal, throat, or saliva result for COVID-19? | Yes<br>No (go to 17.1) |
| 16.3 | Approximately, what was the date of your **first positive test** using a nasal, throat, or saliva sample in 2020? | MM/DD<br>Don’t know |
| 17.1 | Now, thinking about testing for past infection, have you ever had a positive **antibody** test result using a blood sample? | Yes<br>No (GO TO 18) |
| 17.2 | Approximately, when was the date of your **first positive antibody test** using a blood sample in 2020? | MM/DD<br>Don’t know |
| 18.0 | Since March 1, have you experienced any of the following symptoms? (select all that apply) | No symptoms<br>Fever/chills<br>Cough (new onset or worsening of chronic cough)<br>Sore throat<br>Shortness of breath or difficulty breathing<br>Diarrhea<br>Nausea or vomiting<br>Fatigue/tiredness<br>Nasal congestion or runny nose<br>Muscle or body aches<br>New loss in sense of smell or taste<br>Headache (new onset or worsening headache)<br>Other symptoms |
| 19. | When did these symptoms start? (estimate as well as possible) | MM/DD<br>Don’t know |
| 20. | Did you receive healthcare for these symptoms? | Yes<br>No |
| 21. | Were you hospitalized for COVID-19 illness? | Yes<br>No |
| 22. | Do you live in a single unit (e.g., house) or multiunit housing (e.g., an apartment)? | Single unit<br>Multiunit |
| 23. | Number of household members currently in the residence including yourself (resident, family, live-in staff, roommates, and long-term visitors) | Specify (scroll) |
| 24. | What is your height? | Feet<br>Inches |
| 25. | What is your weight? | Weight in pounds |
| 26.1 | Do you have any of the following chronic medical conditions? (choose all that apply) | No conditions<br>Asthma<br>Cerebrovascular disease (affects blood vessels and blood supply to the brain)<br>Chronic kidney disease<br>Chronic liver disease<br>COPD/emphysema/chronic bronchitis<br>Diabetes<br>HIV<br>Hypertension or high blood pressure<br>Immunocompromised condition (e.g., autoimmune disease, solid organ transplant, sickle cell disease)<br>Immune weakening medication or therapy (e.g., cancer treatment)<br>Serious heart conditions, such as heart failure, coronary artery disease, or cardiomyopathies<br>Other chronic conditions |
| 26.2 | Do you currently smoke tobacco or vape nicotine or electronic cigarettes?<br>(Select all that apply) | Yes, current smoker<br>Yes, currently use electronic cigarettes<br>No, former smoker |

| Section | Question/item | Response categories |
|---|---|---|
| | | Never smoked |
| 27. | Since March 1, have you been or are you currently pregnant? | Yes<br>No<br>Don’t know |

**Appendix Table 2.** Sample eligibility criteria

| Included health workplaces | Included health occupations |
|---|---|
| Administrative office | Administrative/business office staff/clerk |
| Ambulatory healthcare/dental office | Diagnostic imaging staff |
| Hospital general inpatient | Dietician/dietary services staff |
| Hospital COVID-19 unit | Engineer/maintenance/mechanic/repair staff |
| Hospital intensive care unit | Environmental services/cleaning staff |
| Hospital surgical unit | Laboratory technologist/technician |
| Hospital other location | Nurse (e.g., CRNA, RN, LPN) |
| Hospital emergency room | Nurse assistant (e.g., CNA, CSA, aide, medication technician) |
| Nursing home COVID-19 unit | Occupational/physical/speech therapist |
| Nursing home nonCOVID-19 unit | Other healthcare* |
| | Pharmacist/pharmacist assistant |
| | Physician (e.g., MD, DO) |
| | Physician assistant |
| | Receptionist/medical assistant/patient registration |
| | Social worker/case manager/counselor |
| | Supervisor/manager |
| **Excluded health workplaces** | **Excluded health occupations** |
| Corrections | Corrections officer |
| Department of Health | Emergency medical technician/paramedic |
| Emergency medical system | Fire fighter/inspector/marshal |
| Fire department | Law enforcement/police officer |
| Law enforcement | Military servicemember |
| Military base | Research/epidemiologist |
| Rhode Island emergency management | |
| Rhode Island alternative hospital set up | |
| Rhode Island remote COVID-19 testing site | |
| Rhode Island state warehouses | |
| Traffic and perimeter control | |

*Includes clinical technician (e.g., cardiac, renal, surgical), dentist/dental hygienist/dental assistant, medical examiner/mortuary technician/forensic personnel, patient aide/technician, phlebotomist/medical technician, recreation specialist/therapist, respiratory therapist, surgical technician/technologist, and security guards and COVID-19 testing personnel in hospital and nursing home settings.

**Appendix Table 3.** Adjusted odds ratios and 95% confidence intervals for seropositivity, Rhode Island, USA, July–August 2020*

| | Hospital personnel n = 9,836 | | Nursing home personnel n = 1,494 | |
|---|---|---|---|---|
| Category | Adjusted odds ratio | 95% CI | Adjusted odds ratio | 95% CI |
| Sex | | | | |
| Female (ref Male) | 0.846 | 0.590–1.213 | **0.657** | **0.458–0.941** |
| Age group | | | | |
| 18–24 y | ref | | ref | |
| 25–34 y | **0.533** | **0.310–0.916** | 1.272 | 0.593–2.732 |
| 35–44 y | 0.461 | 0.205–1.037 | 1.214 | 0.438–3.368 |
| 45–59 y | **0.498** | **0.258–0.961** | 1.608 | 0.684–3.780 |
| 60–64 y | **0.476** | **0.232–0.974** | 1.938 | 0.891–4.214 |
| ≥65 y | **0.258** | **0.106–0.628** | 1.751 | 0.590–5.198 |
| Race/ethnicity | | | | |
| Non-Hispanic white | ref | | ref | |
| Non-Hispanic Black | **2.827** | **1.767–4.521** | 1.597 | 0.837–3.047 |
| Non-Hispanic Asian | 1.155 | 0.583–2.287 | 1.903 | 0.991–3.655 |
| Hispanic | **1.697** | **1.350–2.131** | 1.903 | 0.991–3.655 |
| Other | 1.937 | 0.890–4.215 | 1.520 | 0.636–3.632 |
| Decline | 0.376 | 0.115–1.229 | **2.570** | **1.252–5.276** |
| Exposure to person testing positive for COVID-19 | | | | |
| Co-worker (ref not exposed) | **1.873** | **1.362–2.577** | 1.103 | 0.706–1.723 |
| Household member (ref not exposed) | **11.911** | **8.427–16.835** | **9.373** | **4.750–18.497** |

| Category | Hospital personnel n = 9,836 | | Nursing home personnel n = 1,494 | |
|---|---|---|---|---|
| | Adjusted odds ratio | 95% CI | Adjusted odds ratio | 95% CI |
| Patient, no PPE (ref not exposed) | 1.282 | 0.887–1.853 | 1.055 | 0.537–2.073 |
| Patient, with PPE (ref not exposed) | 1.408 | 0.991–2.001 | **2.185** | **1.256–3.803** |
| Other person (ref not exposed) | **1.626** | **1.018–2.599** | **1.917** | **1.265–2.906** |
| Interpersonal interaction (vs none) | **3.375** | **1.345–8.470** | 1.237 | 0.607–2.522 |
| Housing | | | | |
| Multiunit (ref single family) | 0.911 | 0.671–1.236 | 1.399 | 0.878–2.230 |
| Average frequency of aerosol generating procedures per shift per week | | | | |
| 0 times | ref | | ref | |
| 1–5 times | 1.115 | 0.765–1.624 | 1.589 | 0.866–2.916 |
| 6–10 times | 1.597 | 0.875–2.915 | 1.463 | 0.662–3.235 |
| 11–25 times | 0.713 | 0.364–1.396 | 0.959 | 0.248–3.713 |
| >25 times | 1.239 | 0.699–2.199 | 1.312 | 0.629–2.739 |
| Not applicable | 0.931 | 0.554–1.565 | 1.259 | 0.789–2.008 |
| PPE shortage protocol use | | | | |
| No shortage | 1.229 | 0.672–2.247 | 0.942 | 0.458–1.938 |
| Reuse | 0.546 | 0.284–1.052 | 0.886 | 0.421–1.867 |
| Extended use | 0.662 | 0.421–1.043 | 1.486 | 0.769–2.870 |
| Extended and reuse | 0.838 | 0.556–1.263 | 1.305 | 0.659–2.583 |
| Never used PPE | ref | | ref | |
| N95 respirator/PAPR shortage | | | | |
| Yes | 1.064 | 0.667–1.697 | 0.709 | 0.449–1.119 |
| No shortage | ref | | ref | |
| Not applicable | 1.004 | 0.711–1.418 | 0.574 | 0.298–1.104 |
| Occupation† | | | | |
| Administrative/office staff/clerk | 1.355 | 0.765–2.398 | 1.518 | 0.465–4.959 |
| Diagnostic imaging staff | 1.304 | 0.680–2.500 | NA | NA |
| Dietician/dietary services staff | 0.884 | 0.287–2.717 | 1.623 | 0.579–4.552 |
| Engineer/maintenance staff | 0.942 | 0.308–2.886 | NA | NA |
| Environmental services/cleaning staff | 1.104 | 0.304–4.014 | 1.420 | 0.512–3.937 |
| Laboratory technologist/technician | 1.066 | 0.439–2.593 | NA | NA |
| Nurse (e.g., CRNA, RN, LPN) | **1.660** | **1.073–2.568** | 1.782 | 0.929–3.419 |
| Nurse assistant (e.g., CNA, CSA, Aide) | 1.440 | 0.854–2.427 | **2.055** | **1.057–3.998** |
| Occupational/physical/speech therapist | 1.531 | 0.547–4.284 | ref | |
| Other healthcare | 0.708 | 0.401–1.249 | 0.785 | 0.264–2.329 |
| Other nursing home‡ | NA | NA | 1.741 | 0.637–4.759 |
| Pharmacist/pharmacist assistant | 1.754 | 0.762–4.036 | NA | NA |
| Physician (e.g., MD, DO) | ref | | NA | NA |
| Physician assistant | 0.678 | 0.217–2.118 | NA | NA |
| Receptionist/medical assistant | **2.038** | **1.179–3.522** | NA | NA |
| Social worker/case manager | 0.719 | 0.445–1.162 | **3.284** | **1.119–9.638** |
| Supervisor/manager | 0.996 | 0.513–1.935 | 0.669 | 0.213–2.108 |
| Workplace (ref did not work in this setting)§ | | | | |
| Administrative office | 0.765 | 0.485–1.208 | 0.771 | 0.345–1.724 |
| Ambulatory healthcare/dental office | 0.804 | 0.595–1.086 | – | – |
| Hospital general inpatient unit | 1.039 | 0.739–1.462 | 1.758 | 0.379–8.154 |
| Hospital COVID-19 unit | 1.286 | 0.884–1.870 | 0.198 | 0.035–1.121 |
| Hospital intensive care unit | 0.799 | 0.491–1.299 | NA | NA |
| Hospital surgical unit | **0.612** | **0.410–0.912** | NA | NA |
| Other hospital location | 0.965 | 0.541–1.723 | NA | NA |
| Hospital emergency room | 0.487 | 0.184–1.292 | NA | NA |
| Nursing home COVID-19 unit | **3.946** | **1.392–11.186** | **2.860** | **1.606–5.095** |
| Nursing home non–COVID-19 unit | 0.989 | 0.362–2.701 | 0.808 | 0.508–1.284 |

*The adjusted models were estimated using generalized estimating equations including all variables shown. Bolded adjusted odds ratios and 95% confidence intervals are those for which the 95% confidence interval excludes the value of 1.0. NA, not applicable; NH, non-Hispanic; PAPR, powered air purifying respirator; PPE = personal protective equipment.

†For hospital model, physicians were the referent occupation group. For nursing home model, occupational/physical/speech therapists were the referent occupation group.

‡Includes 4 categories with low sample size: engineer/maintenance staff, pharmacist, receptionist/medical assistant, and physician.

§Workplace was represented by non-mutually exclusive dummy variables entered simultaneously into the model. Participants in workplaces with sample size <30 or with 0% seropositivity were included in the model but the workplace was not entered into the model. Some participants worked in facilities in the other agency category. That is, 84 hospital personnel also worked in nursing home COVID-19 and non-COVID-19 units, and 239 nursing home personnel also worked in hospital administrative offices, COVID-19 units and general inpatient units. Results for these categories are shown in this table, but not in the main manuscript.

**Appendix Table 4.** SARS-CoV-2 seropositivity by resident cases, staff cases, and outbreak status among participants who worked primarily in nursing home settings, Rhode Island, USA, July–August 2020*

| Category | n | Seropositive, n | Seropositive, % |
|---|---|---|---|
| Total | 1,462 | 191 | 13.1 |
| Cases per 100 residents | | | |
| 0 to <2.3 | 400 | 5 | 1.3 |
| 2.3 to <16.3 | 369 | 19 | 5.2 |
| 16.3 to <55.9 | 323 | 58 | 18.0 |
| 55.9 to 83.7 | 370 | 109 | 29.5 |
| Cases per 100 staff | | | |
| 0 to <2.1 | 380 | 5 | 1.3 |
| 2.1 to <11.5 | 346 | 16 | 4.6 |
| 11.5 to <19.9 | 359 | 44 | 12.3 |
| 19.9 to 43.2.7 | 126 | 126 | 33.4 |
| Outbreak status | | | |
| No | 1146 | 128 | 10.1 |
| Yes | 125 | 63 | 33.5 |

*Note: 32 participants (2.1%) worked in facilities for which case and outbreak status data were not available.
Outbreak defined as ≥2 contacts within a facility having active COVID-19 or ≥2 persons with COVID-19 linked outside a case investigation.
Resident and staff case count obtained from Rhode Island Department of Health COVID-19 Data Tracker: Congregate Care: https://ri-department-of-health-covid-19-response-testin-1d583-rihealth.hub.arcgis.com
